# The Dung Beetle Dance: An Orientation Behaviour?

**DOI:** 10.1371/journal.pone.0030211

**Published:** 2012-01-18

**Authors:** Emily Baird, Marcus J. Byrne, Jochen Smolka, Eric J. Warrant, Marie Dacke

**Affiliations:** 1 Department of Biology, Lund University, Lund, Sweden; 2 School of Animal, Plant and Environmental Sciences, University of the Witwatersrand, Johannesburg, South Africa; French National Centre for Scientific Research, France

## Abstract

An interesting feature of dung beetle behaviour is that once they have formed a piece of dung into a ball, they roll it along a straight path away from the dung pile. This straight-line orientation ensures that the beetles depart along the most direct route, guaranteeing that they will not return to the intense competition (from other beetles) that occurs near the dung pile. Before rolling a new ball away from the dung pile, dung beetles perform a characteristic “dance,” in which they climb on top of the ball and rotate about their vertical axis. This dance behaviour can also be observed during the beetles' straight-line departure from the dung pile. The aim of the present study is to investigate the purpose of the dung beetle dance. To do this, we explored the circumstances that elicit dance behaviour in the diurnal ball-rolling dung beetle, *Scarabaeus* (*Kheper*) *nigroaeneus*. Our results reveal that dances are elicited when the beetles lose control of their ball or lose contact with it altogether. We also find that dances can be elicited by both active and passive deviations of course and by changes in visual cues alone. In light of these results, we hypothesise that the dung beetle dance is a visually mediated mechanism that facilitates straight-line orientation in ball-rolling dung beetles by allowing them to 1) establish a roll bearing and 2) return to this chosen bearing after experiencing a disturbance to the roll path.

## Introduction

Upon locating a suitable dung pile, a ball-rolling dung beetle cuts off a piece of dung, shapes it into a ball and rolls it away to a distant location for burial and consumption. While rolling, the beetles move away from the dung pile in a straight line; a remarkable feat given that they do this facing backwards with their head pointing towards the ground. Rolling along a straight path is crucial for dung beetles because it guarantees that they will not return to the dung pile where they risk being attacked by other beetles who, rather than making their own ball, would prefer to capitalise on the work of others [1].

Moving along a straight line, either toward or away from a particular location, is important for survival in many different animals. At first, this does not appear to be a particularly difficult task. However, sensory and motor systems are inherently noisy. This makes it is impossible for any animal (or machine) to move along a straight path without using an external reference, or ‘compass’ [2]. Such a compass could be the Earth's magnetic field, distant landmarks, the position of the sun or the moon or the polarisation pattern they generate in the sky.

Apart from enabling an animal to move along a straight line, the use of an external compass would allow the animal to recover it original direction after experiencing and unintended disturbance. By simply rotating about the vertical axis until the original compass input is restored, an animal could quickly and efficiently return to its preferred bearing [3]. This type of scanning strategy can be observed in sandhoppers [4] and ants [5], [6], [7]. Sandhoppers use oscillating left and right rotations to scan the magnetic field to identify the correct orientation with respect to the land-sea axis [4]. Ants use a combination of rotations and pauses to take snapshots of landmarks [7] and to match these snapshots with the current view [5], allowing them to correct for course deviations when returning to their nest after a foraging journey. Rotations about the vertical axis, during which animals scan the relevant compass cue to locate their desired orientation, therefore appear to be a generalised behaviour in orienting invertebrates.

Unlike sandhoppers and ants, however, the task of the dung beetle is not to orient *towards* a known goal (such as the shoreline, or home), but to *move away* from a specific location (the dung pile) in the most efficient way possible. Previous work has shown that the most important external reference cues for dung beetles are the visual cues present in the sky, such as the sun, the moon, or the pattern of polarised light that forms around them [8], [9], [10], [11]. Although we know the nature of cues used by the beetles to maintain their straight roll path, the mechanism by which they select a roll bearing and regain this bearing after a disturbance remains unclear. Interestingly, just before rolling a new ball away from the dung pile, beetles often perform a characteristic ‘dance’ [8], [11], [12]. During this dance, the beetles climb on top of the ball and rotate about their vertical axis while performing a series of brief pauses, before climbing down and starting to roll. This dance can also be seen during the course of the roll, especially when the roll path has been disturbed [8]. Given the behavioural contexts under which it is often observed, as well as the similarities that it shares with orientation behaviours in other animals, we hypothesise that dance behaviour plays an important role in the straight-line orientation of dung beetles.

Here, we investigate the potential role of the dung beetle dance as an orientation mechanism by exploring the circumstances that elicit this behaviour. In particular, we investigate the probability of dance behaviour when beetles are setting their initial direction (experiment 1), when they have to negotiate obstacles (experiments 2–3), when they are unintentionally displaced (experiments 4–5) and when the visual cues guiding them change unexpectedly (experiment 6). We discuss the implications that our results have for understanding the function of this behaviour. Based on our findings, we conclude that the dung beetle dance is indeed an orientation mechanism that 1) enables the beetles to set a roll bearing and 2) allows them to compensate for large disruptions to their rolling path.

## Analysis

### General

Experiments were performed on the farm “Stonehenge” in North-West Province, South Africa (24.5°E, 24.3°S) using adult beetles of the diurnal ball-rolling species *Scarabaeus* (*Kheper*) *nigroaeneus* (Coleoptera: Scarabaeidae) collected in the local area. Captured beetles were individually marked on their elytra with paint and placed in plastic bins filled with soil, where they could make and roll balls from the fresh cow dung provided. No specific permits were required for the described field studies as *S. nigroaeneus* is not an endangered or protected species. Permission for performing the field studies on “Stonehenge” was obtained from the property owners, Ted and Winnie Harvey, before the experiments commenced.

In the experiments with two conditions (2,3,4), each individual beetle experienced both conditions sequentially and the order of presentation of the experimental conditions was alternated for every second beetle.

### Experiment 1: Dancing at the dung pile

Beetles will often dance just after they have made a ball and just before they are about to roll it away from the dung pile. To quantify the frequency of this behaviour, we placed dung beetles on a pile of dung in the centre of a flat sandy area and filmed them using a video camera mounted overhead. This was repeated with 31 different individuals over two days. The proportion of beetles that performed a distinct dance before rolling away from the dung pile was determined from the video footage. A dance was defined as the beetle climbing on top of its ball and rotating about its vertical axis by more than 90°.

**Figure 1:**
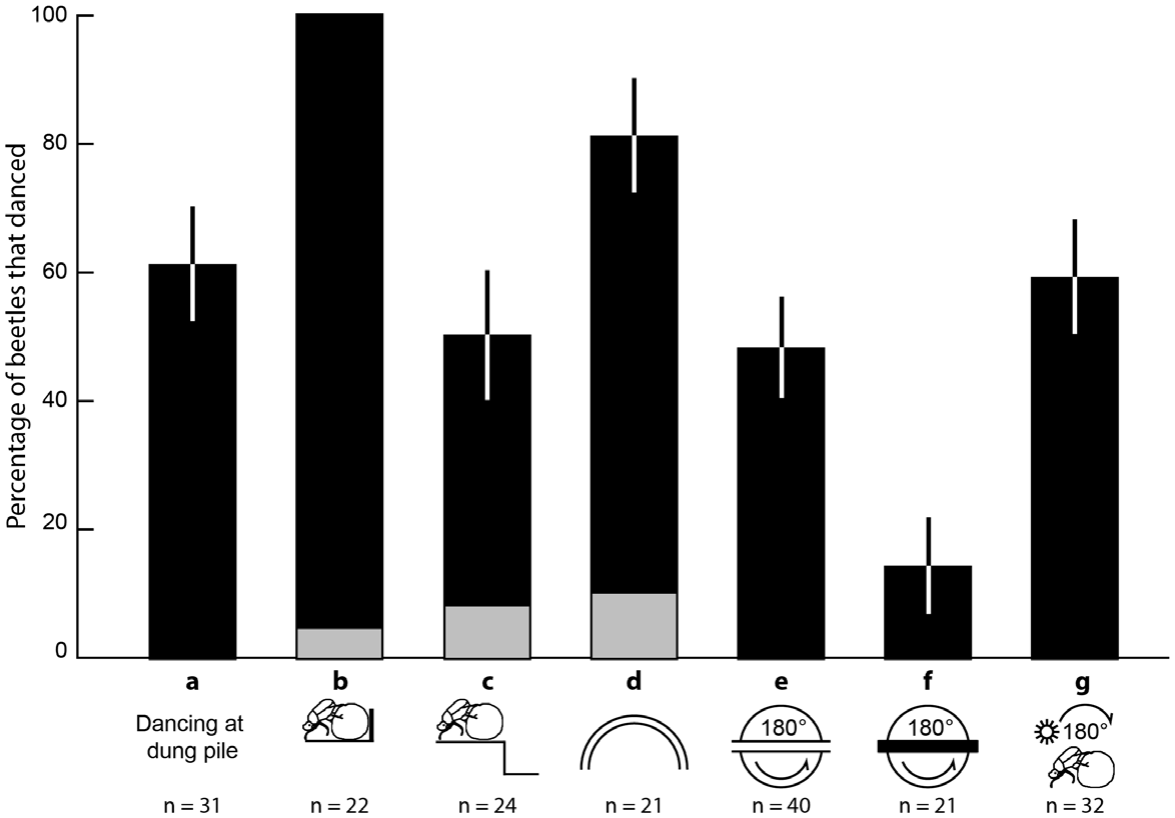
The circumstances that cause dance behaviour in dung beetles. The percentage of beetles that danced under the following conditions: a) at the dung pile, just after making a ball and just before rolling, b) rolling into an obstacle (experiment 2), c) falling off a step (experiment 3), d) rolling off course in a curved tunnel (experiment 4, results from both the 1 m and 1.5 m diameter tunnels are represented with one bar as they were the same), e) 180° rotation with a view of the sky (experiment 5), f) 180° rotation with no view of the sky (experiment 5) and g) 180° reflection of the sun (experiment 6). Grey boxes indicate the results from the control conditions for experiment 2 (b), 3 (c) and 5 (d). Error bars indicate the standard error of the proportion.

Sixty-one per cent (19 of 31) of the beetles performed dances before commencing rolling (figure 1a). On average, dances lasted for 5.7±5.0 s (mean ± std). While the beetles were rotating on top of the ball, they would perform a series of short pauses (3.6±2.6) that lasted 0.46±0.33 s. During the dance, beetles typically rotate 194±114 degrees in either a clockwise or anticlockwise direction and sometimes, but more rarely, in both.

It should be noted that, as beetles make a ball, they constantly move around on top of it and rotate and sometimes make brief pauses during which they hold their head out horizontally. It is therefore possible that the beetles that did not appear to perform a distinct dance were nonetheless scanning the sky and setting a bearing while completing their ball.

### Experiment 2: The effect of obstacles on dance behaviour

When observing a beetle rolling its dung ball away from a fresh dung pile, one notices how the fast, straight and apparently effortless rolling behaviour that occurs on a smooth sandy surface become tortuous and ungainly on rough terrain. Correspondingly, beetles seldom perform dances when rolling on flat ground, whereas they frequently dance when rolling on rough ground.

When rolling on this type of terrain, beetles are often prevented from moving in a straight line by obstacles that force them off course – such as grass tussocks or stones – or by inclines that cause them to lose control of the ball. To investigate if either of these disturbances (obstacles and inclines) are responsible for the increased frequency of dances that is observed on rough ground, we introduced each type of disturbance into the path of beetles rolling on otherwise flat, smooth terrain.

Individual beetles were placed on flat, smooth ground beside a dung ball. Once a beetle had mounted the ball and had established a clear rolling direction (this usually occurred within 25 cm of the starting point), a tunnel (described in detail below) was placed in its path and oriented in line with the rolling direction, so that the beetle could easily enter it without disturbing its roll path. Once in the tunnel, the roll path of the beetles could then be consistently interrupted with a standardised obstacle.

The experimental tunnel consisted of a 5.5 cm wide, 58 cm long, flat wooden floor (covered with sand to prevent the beetles from slipping) and 5 cm high vertical walls made of white opaque plastic that allowed light to enter from the sides, but prevented the beetles from seeing any landmarks. A black plastic flap-door was positioned at the far end of the tunnel. This door was hinged such that it could swing out and upwards when a beetle rolled a ball against it, allowing the ball and beetle to pass through unimpeded. To simulate an obstacle, the door could be fastened shut, so that it did not move when the beetle rolled a ball up against it.

Twenty-two beetles were individually presented with both conditions (flap-door open or closed) in alternating order. We recorded any dances that occurred after a beetle came into contact with the door and the time that it took between these events.

All of the beetles we tested danced within a few seconds (4±2 s, figure 1b, black bar) of hitting an obstacle (the closed flap-door at the end of a tunnel). In contrast, only one beetle danced when it came into contact with the flap-door when it was allowed to swing open (figure 1b, grey bar). Encountering an obstacle that stops the ball from moving thus appears to be a strong cue for dance initiation. It should be noted that all beetles continued to roll straight into the closed door after performing a dance, rather than trying to modify their trajectory so as to avoid the obstacle. The dance thus does not appear to function as a mechanism for identifying obstacles and finding a suitable path around them.

### Experiment 3: The effect of a disturbance on dance behaviour

As in the previous experiment, beetles were allowed to enter a straight tunnel after having freely established a rolling direction. The tunnel was similar to the one used in experiment 2, except that it was open at both ends and divided into two, 83.5 cm long pieces. In the test condition, the second piece of the tunnel was lowered by 5 cm, creating a downward drop in the centre of the tunnel. The drop was large enough to disturb the rolling behaviour of the beetles, but not so large that the beetles lost contact with the ball as they fell. In the control condition, the second piece of the tunnel was raised so that the entire length of the tunnel was level.

Twenty-four beetles were presented with both conditions in alternating order. Any dances that occurred in the second half of the tunnel were recorded (no beetles danced in the first half of the tunnel).

Fifty per cent of beetles (12 of 24) danced after their roll path had been disturbed by the drop (figure 1c, black bar). In contrast, only 8% (2 of 24) danced when the tunnel had no drop (figure 1c, grey bar). Loss of control of the ball that would occur as a result of falling down a sharp incline is thus another circumstance that initiates dance behaviour in dung beetles.

### Experiment 4: The effect of rolling off-course

The results from experiments 2–3 show that beetles are likely to perform a dance when they encounter obstacles or lose control of their ball. Are these events necessary to elicit dance behaviour? In the next series of experiments, we tested if orientation errors – self-induced or passive – are sufficient to generate dances.

To test the effect of terrain-induced, progressive course changes on dance behaviour, we forced beetles to gradually roll off course using semi-circular experimental tunnels. These tunnels were constructed in the same way as those used in the previous experiments, except that they were curved, so that a beetle rolling inside them was forced off its original course by up to 180° (if it rolled to the end of the tunnel). We tested the beetles in tunnels of two different circular diameters (1 m and 1.5 m) to test whether rolling time, rolling distance or accumulated bearing error affected dance behaviour. Beetles were initially placed beside their balls in the centre of a flat wooden disc (42 cm diameter), once they had mounted their ball, performed a dance and started rolling, the curved tunnel was placed at the edge of the disc, such that the beetle entered it in its chosen rolling direction.

Twenty-one beetles were presented with both tunnels in alternating order and with alternating leftwards or rightwards curvatures. We recorded the position of each dance and the angular deviation (the difference between the original rolling bearing and the bearing at the time of dancing) at which it occurred. To control for the effect of the tunnel on dance behaviour, a second group of 21 beetles rolled along a straight tunnel of the same total length (1.6 m) as the 1 m diameter semi-circular tunnel. Any dances that occurred in this tunnel were recorded.

When forced off course in a semi-circular tunnel, 81% (17 of 21) of beetles performed at least one dance (figure 1d, black bar). This result was the same for both the 1 m and 1.5 m diameter tunnels. In contrast, only 10% (2 of 21) danced when rolling along a straight tunnel of the same length (figure 1d, grey bar). This indicates that the likelihood of beetles dancing is increased when the beetles are forced off course by the terrain, even without encountering an obstacle or losing control of the ball.

**Figure 2:**
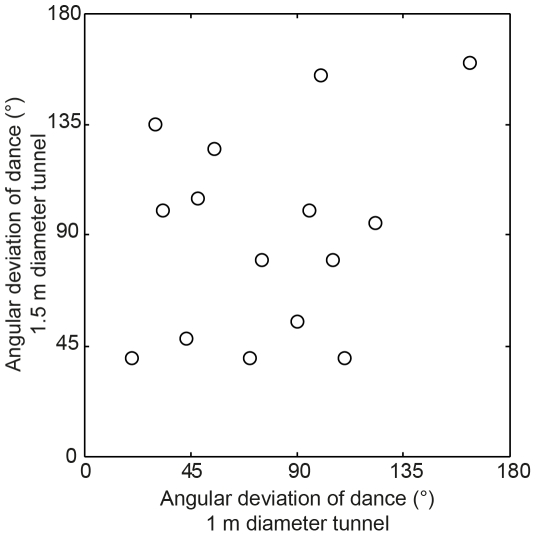
The effect of rolling off course on dance behaviour. The correlation between the angular deviation of dances performed by individual beetles in the 1 m and 1.5 m diameter semi-circular tunnels.

How accurately can beetles measure this accumulated angular error? We recorded the position of each dance and calculated the angular difference, or error, between the roll bearing of the beetle just before it danced and its original roll bearing. Interestingly, these angular deviations are widely spread in both tunnels (figure 2). Furthermore, individual beetles that performed a dance in both tunnels did not dance at the same angular deviation in the two tunnels (pairwise Pearson's linear correlation coefficient, r = 0.28, p = 0.31). These results suggest that even though dances can be triggered by accumulated orientation errors, individual beetles either do not have a set error threshold that they will tolerate before dancing, or simply are not able to measure the error accurately while rolling.

### Experiment 5: The effect of passive course changes

In the previous experiment, beetles were forced to actively roll off course by the walls of the semi-circular tunnel. Under these conditions, beetles could use both tactile cues, created by contact with the walls, and visual cues, such as a change in the celestial compass input, to detect their angular displacement.

In the next experiment, we removed these cues by passively rotating the beetles off their intended course. A straight experimental tunnel (58 cm long) was aligned along the centre of a circular wooden board that could be freely rotated. Individual beetles were placed on the board beside their ball and allowed to roll away in any direction for a short distance. Once the preferred roll bearing of the beetles had been established, the tunnel was aligned in this direction and the beetle was allowed to enter it. When the beetle reached the centre of the tunnel (which was also at the centre of rotation of the circular board), the tunnel was quickly (in under 2 seconds) rotated by 180°, so that the beetle was now rolling in the direction opposite to that in which it had started. We recorded whether the beetle danced after the tunnel rotation and the time taken between the end of the rotation of the tunnel and the dance. A total of 40 beetles were tested.

Forty-eight per cent (19 of 40) of beetles danced after the tunnel rotation (figure 1e). Of these beetles, 95% (18 of 19) changed their rolling direction after dancing. One beetle that did not perform a dance also changed direction after the tunnel rotation.

During the rotation of the tunnel, the beetles had access to both inertial and visual information (from the sky and tall landmarks such as trees and buildings) that could have allowed them to detect the change in orientation. To determine the relative importance of these different cues to the detection of bearing errors, we repeated the experiment when the tunnel was covered with black paper, using a new group of 21 beetles. This prevented the beetles from seeing any celestial or landmark cues. Beetles rolling in this tunnel did not experience complete darkness, as light was still able to enter the tunnel through the opaque white walls. To prevent the beetles from using the light gradient in the tunnel to detect the rotation visually, the experiment was conducted in the shade at mid-day, when the sun was directly overhead. Visual information from the ends of the tunnel was blocked by a swinging flap door at one end (to allow the beetles to enter the tunnel), and a video camera attached to the other end. This camera was used to monitor the beetles' behaviour in the tunnel. Again, we recorded the position and timing of any dance that occurred after the tunnel rotation.

When external visual cues were removed by covering the tunnel, only 14% (3 of 21) performed a dance after being passively rotated (figure 1f) and no beetles changed rolling direction. These results indicate that changes primarily in visual, not inertial, cues are important for eliciting dances in some beetles, even if these changes are not actively generated by self-motion.

### Experiment 6: The effect of changing compass cues on dance behaviour

The aim of this experiment was to test the influence of a change in the position of the sun – *Scarabaeus nigroaeneus*' main compass cue [8] – on dance behaviour. A straight experimental tunnel (83.5 cm long) was placed in the path of the beetle and oriented in its preferred roll direction. Once a beetle had reached the centre of the tunnel, a mirror was used to reflect the image of the sun onto the beetle, shifting the apparent azimuth of the sun by 180° [13]. At the same time, the real sun was shaded from the beetle using a large wooden board. We recorded whether the beetles performed a dance in response to the reflection of the sun and whether they changed their rolling direction.

When the position of the sun changed, 59% (19 of 32) of beetles danced (figure 1g). Of the beetles that danced, 79% (15 of 19) changed their rolling direction by 180°. Due to the constraints of the tunnel, we could not determine the new direction that the beetles wanted to take, as they were all forced to roll back along the tunnel. In contrast, beetles that did not perform a dance did not change direction. These results show that dance behaviour in some dung beetles can be elicited by simply changing the apparent position of the sun in the sky. This highlights the importance of the sun as a compass cue in these beetles and suggests an important role for the dance as a mechanism for course correction.

## Discussion

Here, we show that most beetles perform a characteristic dance – in which they climb on top of the ball and rotate about their vertical axis – before rolling away from the dung pile. The likelihood of a dance being performed increases when the beetles encounter an obstacle or lose control of the ball. Our results also show that it is not only the physical disruption that beetles experience during a disturbance that causes them to initiate a dance. Dancing can also be elicited by changing the orientation of the ball rolling beetles relative to the position of the celestial visual cues, or just the apparent position of the sun. In light of these results, we conclude that the dance is a behavioural mechanism that facilitates straight-line orientation in ball-rolling dung beetles by allowing them to 1) establish a roll bearing and 2) to return to this chosen bearing if their roll path is disrupted unintentionally.

For dung beetles, celestial cues are the most important compass reference for maintaining a constant bearing while rolling [8], [9], [10], [11]. This preferred roll bearing is not hardwired but, instead, changes after a beetle makes a new ball [14]. Beetles thus ‘reset’ their preferred compass bearing sometime between making a ball and rolling it away from the dung pile. Here, we show that, just before rolling their ball away from the dung pile, most beetles perform a characteristic dance during which they rotate about their vertical axis while making a number of brief pauses. This behaviour is strikingly similar to the orientation behaviour observed in ants, which use rotations interspersed with pauses to locate and take snapshots of relevant landmark cues [5], [6], [7].

We propose that, during the initial dance at the dung pile, beetles store a compass reading of the celestial cues as they appear along the preferred roll orientation. They then try to match their stored compass reading to the cues they see while rolling, thereby allowing them to move away from the dung pile in a straight line. This hypothesis is supported by the result that most of the beetles that danced in response to a 180° shift in the sun's position also changed their roll bearing. This behaviour would be expected if the beetles were trying to hold constant the position of the sun in the visual field. The aim of future investigations will be to test this hypothesis by examining the fine details of the dance and attempting to relate these details to the roll bearing taken by the beetle.

In addition to enabling the beetles to roll in a straight line, another advantage of memorising a compass direction is that it would allow the beetles to regain their original orientation when the difference between the original compass bearing and the current bearing becomes too large. If rolling beetles were trying to match the current compass reading with a stored compass reading, then any disruption to the set course would be likely to create an error between the two. Here, we have shown that dances are elicited when beetles experience large disruptions to their roll path or unintended course deviations. The most efficient way to correct for such errors would be for the beetle to scan the environment – by rotating about its vertical axis – until the compass reading once more matches the reading that the beetle stored when it first started rolling. This appears to be exactly what the beetles do after experiencing a disturbance.

It is interesting to note that most beetles dance when they are being forced to roll progressively off course, but that these dances do not occur at the same angular deviation from the original bearing. Different beetles (and even the same beetle rolling in the different curved tunnels) perform dances at very different angular deviations. This is perhaps due to the fact that, while rolling, the head of the beetle is tilted downwards and is constantly moving, making it difficult to detect and respond to errors consistently.

If the function of the dance is to take a compass reading of the celestial cues or find a match between this snapshot and the current view of the sky, then why do beetles climb on top of their ball to scan the sky, instead of staying on the ground? Climbing on top of the ball would have several advantages. Firstly, getting higher would maximise the beetles' chances of getting a good view of the sky. This would be especially useful in grassy areas where the view of the sky from the ground may be occluded by obstacles. Another advantage of climbing on top of the ball would be that it would allow the beetles to perform the rotation on a single plane above the ground, where the ball would not obstruct the field of view. A third advantage of getting on top of the ball would be that it would allow the beetle to easily defend its ball against other beetles that might try to steal it while it is otherwise engaged in an orientation behaviour. Given that beetles rolling on rough ground perform their first dance when they are very close to the dung pile, this defensive approach would be necessary, due to the fierce competition for dung balls near the dung pile.

Thus far, we have focussed on the conditions that elicit dance behaviour in dung beetles. However, instances of non-dancing occur in every situation presented here, with the exception of experiment 2, in which all beetles that rolled into the barrier performed a dance. In the natural context of making balls at the dung pile, 39% of beetles did not perform a well-defined dance, but we cannot exclude the possibility that these beetles were nonetheless taking compass reading cues while they were making their balls. In the remaining experiments, the proportion of non-dances varies from a minimum of 19%, when the beetles are forced to roll off course (experiment 4) and a maximum of 52%, when the beetles are passively rotated (experiment 5). One possible explanation for these results – at least for the experiments in which the beetles fell off a drop (experiment 3) – is that the non-dancers landed in exactly the same orientation that they had while rolling, making it unnecessary to reorient using a rotation. This explanation alone, however, does not adequately account for the non-dances that occur when the beetles are either actively or passively forced off course (experiments 4 and 5, respectively). The result that 41% of beetles did not dance when the sun was reflected by 180° (experiment 6) suggests that these beetles were not orienting with respect to the sun but were instead relying on a celestial cue that remained unchanged during the experiment, such as the pattern of polarised light or the light intensity gradient. Exploring the reasons for non-dancing by focussing on the behaviour of non-dancers before and after encountering a disturbance will help to provide a better understanding of the mechanisms of beetle orientation and will provide an informative focus for future studies.

### Conclusions

Unlike many other animal navigators, the task of the dung beetles is not to find their way back to a familiar location after a foraging trip. Instead, foraging dung beetles need to roll from a known location to an unknown destination in the most direct and efficient manner possible, which is in a straight line. Here, we propose that the characteristic dance that dung beetles perform before rolling away from the dung pile, and after encountering a disturbance while rolling, is an orientation mechanism that allows beetles to set an initial roll bearing, and to regain this original bearing if they experience an unintentional disturbance.
